# Free subsets in internally approachable models

**DOI:** 10.1007/s00153-024-00947-0

**Published:** 2024-10-22

**Authors:** P. D. Welch

**Affiliations:** School of Mathematics, University of Bristol, Fry Building, Woodland Road, Bristol, BS8 1UG England

**Keywords:** Free subsets, Inner models, Measurability, Pcf theory, 03E04, 03E20, 03E45, 03E55

## Abstract

We consider a question of Pereira as to whether the characteristic function of an internally approachable model can lead to free subsets for functions of the model. Pereira isolated the pertinent *Approachable Free Subsets Property* (AFSP) in his work on the ${\text {pcf}}$-conjecture. A recent related property is the *Approachable Bounded Subset Property* (ABSP) of Ben-Neria and Adolf, and we here directly show it requires modest large cardinals to establish:

**Theorem** *If ABSP* holds for an ascending sequence $\langle \aleph _{n_{m}} \rangle _{m}$ $( n_{m} \in \omega )$ then there is an inner model with measurables $\kappa < \aleph _{\omega }$ of arbitrarily large Mitchell order below $\aleph _{\omega }$, that is: $\sup \left\{ \alpha \mid {\exists }\kappa < \aleph _{\omega } o ( \kappa ) \ge \alpha \right\} = \aleph _{\omega }$. A result of Adolf and Ben Neria then shows that this conclusion is in fact the exact consistency strength of ABSP for such an ascending sequence. Their result went via the consistency of the non-existence of continuous tree-like scales; the result of this paper is direct and avoids the use of PCF scales.

## Introduction

To introduce the results we first state some definitions. Recall that *N* being an *internally approachable* substructure (of length $\tau$) means, to be concrete, and to be specific to our situation rather than completely general, that $N= \bigcup _{\iota < \tau } N_{\iota }$ for some $\langle N_{\iota } | \iota < \tau \rangle$ which is a continuous chain of substructures of $N_{\iota } \prec {\langle }H ( \theta ), \in , \langle F_{n} \rangle _{n< \omega },\ldots {\rangle }$ - continuous meaning in turn that $\langle N_{\xi } \mid \xi \le \iota \rangle \in N_{\iota +1}$ and ${\text {Lim}} ( \zeta ) {\,\longrightarrow \,}\bigcup _{\iota < \zeta } N_{\iota } =N_{\zeta }$, for $\iota< \zeta < \tau$. Our internally approachable substructures will always be of length some $\tau$ of uncountable cofinality. The functions $F_{n}$ are all finitary functions of the form $F_{n}:\, ^{m_{n}} \! H ( \theta ) {\,\longrightarrow \,}H ( \theta )$. Typically, and as will always be the case for us, amongst the $F_{n}$ will be a set of $\text {Skolem}_\text {Function}$ functions for the structure $\langle H ( \theta ), \in \rangle$ (and more). We shall often think of this as a single function $F:\, ^{< \omega }H ( \theta ) {\,\longrightarrow \,}H ( \theta )$. For $C { \,\subseteq \, } \theta$, we say that “*C* *is free for* *F*” to mean that ${\forall }\xi \in C( \xi \notin F$“$[C {\backslash }\{\xi \}]^{< \omega } )$. Thus for our *F* this means in particular that members of *C* are not definable over the structure from other members of *C*. For $N \prec {\langle }H ( \theta ), \in , \langle F_{n} \rangle _{n< \omega } {\rangle }$ we shall say that for $X { \,\subseteq \, } H ( \theta )$, that *X* is *free over* *N* if for any *n* and all $\zeta \in X, \zeta \notin F_{n}$“$( N \cup X {\setminus } \{ \zeta \} )$.

Cardinals $\lambda$ with $H ( \lambda )$ possessing free subsets have been studied for some years: we say $\lambda$ *has the free subset property* if for any algebra $\mathcal {H}$ on $\lambda$ or some larger structure $\mathcal {H}$ extending $H ( \lambda )$, then $\mathcal {H}$ has a free subset $X { \,\subseteq \, } \lambda$ which is cofinal in $\lambda$. In [8], a model is constructed by forcing over a model with infinitely many measurable cardinals in order to have infinite free subsets for any $F: [ \aleph _{\omega } ]^{< \omega } {\,\longrightarrow \,}\aleph _{\omega }$. In [3] it was shown that the assumption that $\aleph _{\omega }$ is free in this sense is equiconsistent with the existence of a single measurable cardinal. Pereira in ([6, 7]) in his work on Shelah’s pcf conjecture isolated the following strengthening of this free subset property in the specific situation of the characteristic function of internally approachable substructures whose range provides a free set for the substructure.

We use the notation that if $N \prec H ( \theta )$ and $\tau \in {\text {Card}} \cap N$, then $\chi _{N} ( \tau ) =_{{\text {df}}}\sup N \cap \tau$. If we have a sequence of such cardinals $\langle \tau _{\alpha } \rangle _{\alpha < \delta }$ from *N*, then we shall abbreviate $\chi _{N} ( \alpha ) = \chi _{N} ( \tau _{\alpha } )$ when there is no danger of confusion. (For this note, $\delta$ is mostly $\omega$ and $\alpha$ is some *n*.) If $Y { \,\subseteq \, } H ( \theta )$ and $N \prec H ( \theta )$ then we write *N*[*Y*] for the skolem hull in $H ( \theta )$ of *N* together with *Y*. Thus $N [ Y ] \prec H ( \theta )$ also.

### Definition 1

(Pereira cf. [6], or [7]) The *Approachable Free Subset Property* (AFSP) *for* $\aleph _{\omega }$ states that for every internally approachable $N \prec {\langle }H ( \theta ), \in , {\langle }F_{n} {\rangle }_{n< \omega } {\rangle }$, the latter any extension of $\langle H ( \theta ), \in \rangle$, of length $\omega _{l}$, for some $l< \omega$ and some large $\theta$, if $\chi _{N} (m) =_{{\text {df}}}\sup (N \cap \omega _{m} )$ for $\omega>m>k$ (for some k) then there is an infinite subset $C'$ of $C =_{{\text {df}}}\{\chi _{N} (m) | k<m< \omega \}$ which is *free over* *N*. That is as above, for any *m* and *n* with $\chi _{N} (m) \in C'$, then $\chi _{N} (m) \notin F_{n}$“$\,( N \cup C' {\setminus } \{ \chi _{N} (m) \} )$.

Pereira showed that if the negation of AFSP at $\aleph _{\omega }$ was provable in ZFC, then the ${\text {pcf}}$-conjecture (that $| {\text {pcf}} ( A ) | \le | A |$ for any progressive set of regular cardinals *A*) was also so provable. The well-known consequence of this for cardinal arithmetic by Shelah’s work is then established: that if $\aleph _{\omega }$ was a strong limit cardinal, then $2^{\aleph _{\omega }} < \aleph _{\omega _{1}}$. So it was clearly of interest at that time (2008) to investigate it. Pereira showed ([6], Sect. 6.3) that AFSP cannot hold in the Koepke model of [3].

Note: we may similarly define AFSB for $\tau = \sup {\langle }\tau _{n}{\rangle }_{n<\omega }$ the latter an ascending sequence of regular cardinals ${\langle }\tau _{n}{\rangle }_{n<\omega }$.

We need to assume some familiarity with the *core model* *K* as described in [9], and write it as $K=L [E^{K} ]$ where $E^{K}$ codes, as usual here, a sequence of both partial and full measures and extenders $E_{\nu }$ for certain $\nu \in {\text {On}}$. It is usual to write $K_{\kappa }$ *etc.*  for $L_{\kappa } [E {\upharpoonright }\kappa ]$. In the sequel we shall consider extender models where $E_{\nu }$ may be an extender on some critical point $\kappa$ much smaller than the index $\nu$. However these models will be of the ‘non-overlapping extender’ type, and indeed we shall not be considering models with, *e.g.*  a strong cardinal. We shall let $o^{K} ( \alpha )$ denote the Mitchell order of measurability of the ordinal $\alpha$ in *K* thus $o^{K}  ( \alpha )$ is the order type of $\left\{ \nu \mid \, E_{\nu }^{K} \right.$ is a full measure $\wedge \, \, {\text {crit}}$($E^{K}_{\nu } ) = \alpha \}$.

The interested reader is encouraged to look at [1] where a more detailed history and background to the AFSP and neighbouring properties is discussed in detail.

In 2008 we had sketched in an unpublished note an argument for the following:

$$(*)$$$${\text {Con}} ( {\text {ZFC}} + {\text {AFSB}} $$ *for* $$\aleph _{\omega } ) \Rightarrow $$ $${\text {Con}} ( {\text {ZFC}} + $$ *For any* $$k \ge 1$$ *and for arbitrarily large *$$m>k \{\alpha < \omega _{m} | o^{K} ( \alpha ) \ge \omega _{k } \}$$ *is stationary*$$)$$.

Then very recently Omer Ben-Neria and Dominik Adolf [1] showed by a forcing construction the reverse of the consistency implication of the above theorem. Consequently the result of that note proving $(*)$ became more germane. However when we came to write out the proof more closely we noticed a gap, which we are unable to bridge. However we can still get this conclusion from a *prima facie* slightly stronger principle of theirs:

### Definition 2

(The Approachable Bounded Subset Property - ABSP).

Let $\langle n_{m} \rangle _{m< \omega }$ be an ascending sequence from $\omega$. The ABSP for ${\langle }\aleph _{n_{m}} {\rangle }_{m< \omega }$ states that for every internally approachable $N \prec {\langle }H ( \theta ), \in , {\langle }F_{n} {\rangle }_{n< \omega } {\rangle }$, the latter any extension of $\langle H ( \theta ), \in \rangle$, of length $\omega _{k}$ for some $0<k< \omega$ and some $\theta > \omega _{\omega }$, if $\chi _{N} (m) =_{{\text {df}}}\sup (N \cap \omega _{n_{m}} )$, then for some $n_{0} < \omega$, setting $X= \{ \chi _{N} ( m ) \mid m \ge n_{0} \},$ for any $m \ge n_{0}$

$$\chi _{N} ( m ) = \chi _{N [ X \backslash \{ \chi _{N} ( m ) \} ]} ( m ).$$

To paraphrase: if we take the skolem hull of the original structure *N* together with the range of this characteristic $\chi$-function defined from $\langle n_{m} \rangle _{n_{0} \le m< \omega }$, with one point from that range omitted, this ensures that the skolem hull does not increase below the next cardinal above that dropped point. (The authors of [1] call this *Bounded Appending*.)

### Remark 1

- *ABSP* for ${\langle }\aleph _{n_{m}} {\rangle }_{m< \omega }$ implies that for $m \ge n_{0}$ and *X* in the above definition, and for any $F \in N$, that:
- *F*“$\,( N \cup X {\setminus } \{ \chi _{N} (m) \} ) \cap [ \chi _{N} ( m ), \omega _{n_{m}} ) = {\varnothing }$ and in particular
- $$\chi _{N} (m) \notin F$$“$$\, ( N \cup X {\setminus } \{ \chi _{N} (m) \} )$$.
- Thus if ABSP for ${\langle }\aleph _{n_{m}} {\rangle }_{m< \omega }$ holds then *a fortiori* *AFSP* for $\aleph _{\omega }$ holds (*cf.* Definition 1).
- Similarly we define *ABSP* in exactly the same way for ${\langle }\tau _{{m}} {\rangle }_{m< \omega }$ any ascending sequence of regular cardinals, rather than just an infinite subset of the $\aleph _{n}$. We shall use this in the sequel.

With hindsight the previous argument for $(*)$ actually showed (or can be read as having showed, when stated with Adolf and Ben Neria’s definition):

### Theorem 1

${\text {Con}} ( {\text {ZFC}} + {\text {ABSP}}$ for ${\langle }\aleph _{n_{m}} {\rangle }_{m< \omega }$, for some infinite $\left. \{ n_{m} \}_{m,\omega } { \,\subseteq \, } \omega \right) {\,\Rightarrow \,}$

$${\text {Con}} ( {\text {ZFC}} + $$For any $$k \ge 1$$, for arbitrarily large $$m>k \{\alpha < \omega _{m} | o^{K} ( \alpha ) \ge \omega _{k } \}$$ is stationary$$)$$.

The reason for going from ${\text {AFSP}}$ in $(*)$ to ${\text {ABSP}}$ in Theorem 1 is to get the extra closure in the hulls at (3) in Sect. 2 below. See the discussion in Sect. 3 at open Question 1.

Now with the following it turns out that the conclusion of Theorem 1 (or $(*)$) was at the right level for an an equiconsistency:

### Theorem 2

(Adolf, Ben-Neria [1]) If there exists a cardinal $\tau = \sup \{ \tau _{n} \}_{n< \omega }$ with $\tau _{n}$ an increasing sequence of regular cardinals, with the set of Mitchell orders $\{ o ( \tau _{n} ) \mid n< \omega \}$ unbounded in $\tau$, then there is a forcing extension in which ${\text {ABSP}}$for some infinite set ${\langle }\aleph _{n_{m}} {\rangle }_{m< \omega }$ holds (and hence so does ${\text {AFSP}}$ at $\aleph _{\omega }$).

In fact [1] (*cf.*their Cor. 8) they show more through the following:

### Theorem 3

(Adolf, Ben-Neria [1], Cor. 8) The following are equiconsistent:

1. There exists an ascending sequence of regular cardinals $\langle \tau _{n} \rangle _{n< \omega }$ for which the ${\text {ABSP}}$ holds for $\langle \tau _{n} \rangle _{n< \omega }$.
2. here exists an ascending sequence of regular cardinals $\langle \tau _{n} \rangle _{n< \omega }$ for which the ${\text {AFSP}}$ holds for $\langle \tau _{n} \rangle _{n< \omega }$.
3. There exists an ascending sequence of regular cardinals $\langle \tau _{n} \rangle _{n< \omega }$ for which the product $\prod _{n} \tau _{n}$ does not carry a continuous tree-like scale.
4. There exists a cardinal $\lambda$ such that the set of Mitchell orders $\{ o ( \mu ) \mid \mu < \lambda \}$ is unbounded in $\lambda$.

This establishes the equiconsistency of ABSP at $\aleph _{\omega }$ with AFSP at $\aleph _{\omega }$, and then with this the hypothesis of the last Theorem,

But the equiconsistency argument establishing (4) is done in the order stated and goes first through (3) as an intermediary property of the non-existence of certain continuous scales. Our next theorem gives a direct argument for the lower bound. Let “There is no inner model with a $\kappa$ with $(o(\kappa )=\kappa ^{++}))$” be abbreviated as $\lnot IM(o(\kappa )=\kappa ^{++})$.

### Theorem 4

$$( {\text {ZFC}} )$$ $$(\lnot IM(o(\kappa )=\kappa ^{++}))$$ $$ {\text {ABSP}}$$ for $${\langle }\aleph _{n_{m}} {\rangle }_{m< \omega }$$, for some $$\{ n_{m} \}_{m} { \,\subseteq \, } \omega $$ implies that for all sufficiently large $$m>k, \{\alpha < \omega _{n_m} | o^{K} ( \alpha ) \ge \omega _{k } \}$$ is stationary$$)$$.

However in Sect. 2 we shall not prove the last theorem, but rather give a proof of the direct implication in Theorem 1*via * the following which subsumes several of our results under one heading. Recall that there is no sharp for an inner model with a strong cardinal, is known as the assertion: $\lnot O^{{\text {\P }}}$: “not-oh-pistol“”.

### Theorem 5

($\lnot O^{{\text {\P }}} )$ Let $\langle \tau _{n} \rangle _{n< \omega }$ be an increasing sequence of regular cardinals, for which ${\text {ABSP}}$ holds.

(i) If the $\tau _{n}$ are inaccessible cardinals in *K* then for any $k\ge 1$, for all sufficiently large *m*, either $\{\alpha < \tau _{m} | o^{K} ( \alpha ) \ge \tau _{k } \}$ is stationary below $\tau _{m}$ or there is $\lambda _{m}<\tau _{m}$ with $o^{K}(\lambda _{m})\ge \tau _{m}$.
(ii) If additionally in (i), for all $\gamma < \tau =_{{\text {df}}}\sup _{n} \tau _{n}$ we have ${\text {cf}} ( \gamma ) = {\text {cf}}^{K} ( \gamma )$ then the second alternative holds: for a tail of the $\tau _{m}$, there is $\lambda _{m} < \tau _{m}$ with $\lambda _{m}$ strong up to $\tau _{m}$.
(iii) If the $\tau _{n}$ are successor cardinals in *K*, with $\tau _{n} = \lambda _{n}^{+K}$ for $\lambda _{n}$ *K*-cardinals, then
  $$\{ \alpha \mid E^{K}_{\alpha } \text{ is } \text{ an } \text{ extender } \text{ with } \mathop {crit} ( E^{K}_{\alpha } ) < \lambda _{m} \}$$
  is unbounded in $\tau _{m}$ for all sufficiently large $\tau _{m}$.

### Remark

In (i) this naturally implies the consistency of a cardinal $\lambda$ such that the set of Mitchell orders $\{ o ( \mu ) \mid \mu < \lambda \}$ is unbounded in $\lambda$. If we had built the core model assuming $\lnot IM( (o(\kappa )=\kappa ^{++})$ for example, then only the first alternative could have held.

Note in (iii) that this implies there are infinitely many *n* and $\nu _{n}$, with $\lambda _{{n-1}}<\nu _{n} < \lambda _{n}$ and $( o ( \nu _{n} ) \ge \nu ^{+ +}_{n} )^{K}$.

## Proof of Theorem 5

### Proof

(i) The $${\text {ABSP}}$$ for $${\langle }\tau _{n} {\rangle }_{n< \omega }$$ states that for every internally approachable $$N \prec {\langle }H ( \theta ), \in , {\langle }F_{n} {\rangle }_{n< \omega } {\rangle }$$, the latter any extension of $$\langle H ( \theta ), \in \rangle $$, of length $$\tau _{k}$$ for some $$0<k< \omega $$ and some $$\theta > \tau =_{{\text {df}}}\sup _{n} \{ \tau _{n} \}$$, if $$\chi _{N} (m) =_{{\text {df}}}\sup (N \cap \tau _{m} )$$, then for some $$n_{0} < \omega $$, setting $$X= \{ \chi _{N} ( m ) \mid m \ge n_{0} \},$$ for any $$m \ge n_{0}$$$$\chi _{N} ( m ) = \chi _{N [ X \backslash \{ \chi _{N} ( m ) \} ]} ( m )$$.

We suppose that the conclusion fails, *i.e.*  that in *K* and for an infinite set $Q { \,\subseteq \, } \omega$ we have for $m\in Q$ that there is no $\lambda _{m}<\tau _{m}$ with $o^{K}(\lambda _{m})\ge \tau _{m}$ and that we can find closed and unbounded sets $G_{m} { \,\subseteq \, } \tau _{m}$ and with no $\alpha \in G_{m}$ having $o^{K} ( \alpha ) \ge \tau _{k}$. Fix least such *Q* and ${\langle }G_{m}|m\in Q{\rangle }$, in a fixed wellorder of ${\mathcal {P}}( \tau )$, and have them, and the wellorder, as part of our algebra on $H ( \theta )$.

We first note: we shall take the functions $\langle f_{n} \rangle _{n}$ of our algebra to include an *F* to code a complete set of skolem functions for $\mathcal {K} =_{{\text {df}}}\langle K_{\tau }, \in , E^{K} {\upharpoonright }\tau \rangle$ and we note that with $\mathcal {H}= {\langle }H ( \theta ), \in , Q,{\langle }{G_{m}}{\rangle }_{m\in Q}, {\langle }f^{n} {\rangle }_{n< \omega }, \ldots {\rangle }$, both $\mathcal {K}$,*F*, and ${\langle }G_{m}{\rangle }_{{m\in Q}}$ are definable and so in $N_{0} \prec \mathcal {H}$ for some, or any, appropriate regular $\theta > \tau ^{+}$. Let *N* be internally approachable, as witnessed by an $\tau _{k}$-chain of models ${\langle }N_{\iota } | \iota < \tau _{\kappa } {\rangle }$ with $N_{\iota } \prec \mathcal {H}$, and with *N* their union. For $X { \,\subseteq \, } H ( \theta )$ we let *N*[*X*] be the skolem hull in $\mathcal {H}$ of $N \cup X$, which we shall also write as ${\text {SH}}^{\mathcal {H}} ( N \cup X )$.

We recall first some properties of mice. $\square$

### Lemma 1

(The Dodd-Jensen Lemma) (eg., [5] 3.41 or [9] Lemma 4.3.9) Suppose that *M* is a mouse and $j:M \rightarrow N$ is an iteration by extender ultrapowers of *M* to *N*. Let $k:M \rightarrow N$ be any other $\Sigma _{0}$-preserving map. Then:

(i) *k* is cofinal into *N*;
(ii) $${\forall }\alpha \in M ( j ( \alpha ) \le k ( \alpha ) )$$;
(iii) The iteration whose map is *j* does not drop in degree, nor does it involve a truncation, at any stage.

- Fact 1: There is a canonical prewellordering of mice and weasels (the latter are class-sized mice) so that $M \le ^{*} N$ iff in the coiteration of (*M*, *N*) to a pair $( M_{\infty },N_{\infty } )$ which are comparable hierarchies, then $M_{\infty }$ is an initial segment (not necessarily proper) of $N_{\infty }$. We let $=^{*}$ denote the corresponding equivalence class (*cf.*[9]).
- Fact 2: If $M \le ^{*} N$ are mice, then in the coiteration of (*M*, *N*) to a comparable pair $( M_{\infty },N_{\infty } )$ there can be no truncation on the *M*-side of the iteration.

We shall use the following consequence of a strengthening of the Covering Theorem for *K* below $O^{\P }$, due to Cox:

### Theorem 6

(Cox [2]) Suppose $\lnot O^{{\text {\P }}}$. If $\gamma \ge \omega _{2}$ is regular in *K* and $\omega< {\text {cf}}^{V}(\gamma ) <|\gamma |^{V}$, then $o^{K}(\gamma ) \ge {\text {cf}}^{V}(\gamma )$.

Turning back to the proof of the Theorem, by the construction of *N* as an internally approachable chain of length $\tau _{k}$ we have:

(1) *For *$$k+1 \le m< \omega $$, $${\text {cf}}(\chi _{N} (m)) = {\tau _{k}}$$.

By ABSP for $\langle \tau _{n} \rangle _{n< \omega }$, let $n_{0} < \omega$ be such that the Bounded Appending Property holds for *N* with respect to the set $X =_{{\text {df}}}\{ \chi _{N} ( m ) \mid n_{0}<m< \omega \}$, thus for any $n \ge n_{0}$ $\chi _{N} ( n ) = \chi _{N [ X \backslash \{ \chi _{N} ( n ) \} ]} ( n )$. In other words, adding in all the ordinals of *X* to *N*, with $\chi _{N} ( n )$ excepted, does not increase the supremum of the Skolem hull below $\tau _{n}$.

For $r \in \omega$, we set $\pi _{r}: {\text {SH}}^{\mathcal {K}} [ X {\setminus } \chi _{N} ( r ) ] {\leftrightarrow }K^{r}$ to be the transitive collapse map.

(2) *Claim*: *There is a natural number p* *so that for any* $r,s \ge p$*then* $K {^{r}} = ^{*} K^{s}$.

**Proof: **of (2). Note that on general grounds (going back to the Dodd-Jensen Lemma) $r \le s$ implies that $K^{s} \le ^{*} K^{r}$ as $\pi _{r} \circ \pi ^{-1}_{s}:K^{s} {\,\longrightarrow \,}K^{r}$ is a $\Sigma _{0}$, in fact fully elementary, embedding. (Were $K^{r} <^{*} K^{s}$, then in the coiteration of $( K^{s},K^{r} )$ to $( K_{\infty }^{s},K_{\infty }^{r} )$ *via * maps $\pi ^{s}:K^{s} {\,\longrightarrow \,}K^{s}_{\infty }$, and $\pi ^{r}:K^{r} {\,\longrightarrow \,}K^{r}_{\infty }$, we should have either that $K^{r}_{\infty }$ is necessarily a proper initial segment of $K^{s}_{\infty }$ or else at some stage there was a truncation on the $K^{s}$ side. Letting $k= \pi ^{r} \circ ( \pi _{r} \circ \pi ^{-1}_{s} )$, we have that the first alternative does not happen, since $k:K^{s} {\,\longrightarrow \,}K^{s}_{\infty }$ is a $\Sigma _{0}$-preserving map total of $K^{s}$ into $K^{s}_{\infty }$ which is not cofinal; this contradicts (i) of the Lemma 1. The second alternative is ruled out by (iii) of Lemma 1.)

If the *Claim* failed there would be an ascending sequence $r_{n}<r_{n+1} < \omega$, and then a corresponding $<^{*}$ descending sequence of transitive collapse models $K^{r_{n}}$ with $K^{r_{n+1}} <^{*} K^{r_{n}}$ which contradicts the wellfoundedness of the mouse order at Fact 1. $\square$

To facilitate our notation let $q=_{{\text {df}}}\min Q{\backslash }\max \{k+1, p\}$ and $D= \{ \chi _{N} ( n ) \mid n \ge q \}$ and also let $x_{0}<x_{1} < \cdots$ enumerate *D* in ascending order. We set $\pi _{D} =_{{\text {df}}}\pi _{q}$, and $K_{D} =_{{\text {df}}}K^{q}.$

$$\bullet $$ We note that the ABSP implies, *a fortiori*, also that *X* is free for *F*, and then so is the above subset *D*. Moreover if we define $$\overline{D }$$ *via * $$\pi _{D}^{-1}$$“$$\overline{ D } =D$$ then $$\overline{D }$$ is free for $$\overline{F}$$ $$=_{{\text {df}}}$$$$\pi _{D}$$“*F* in $$K_{ D}$$, where $$\overline{F }$$ thus appropriately encodes a set of complete skolem functions for $$K_{{D }}$$.

Let ${\langle }\bar{x}_{i} {\rangle }_{i< \omega }$ enumerate $\overline{ D }$ with $\pi _{D} ^{-1}( \bar{x}_{i} ) =x_{i}$.

(3) *Claim*: *Let* $\alpha <x_{0}$, *then* $\sup N [ \alpha \cup D \backslash \{ x_{0} \} ] \cap \tau _{q} =x_{0}$.

### Proof

We note first that $\sup N [ D \backslash \{ x_{0} \} ] \cap \tau _{q} =x_{0}$ by ABSP. Then this is the usual argument about functions in hulls: as *N* is unbounded in $x_{0}$ we may assume $\alpha \in N$. Let $f \in N$, be such that $f \left( \gamma , \vec {z} \right) <\tau _{q}$ for any $\gamma <x_{0}$, and any $\vec {z}$, and let $\vec {x} \in \, ^{< \omega } ( D {\setminus } \{ x_{0} \} )$ be of the length of $\vec {z}$. Then $\sup \left\{ f \left( \gamma , \vec {x} \right) \mid \gamma < \alpha \right\}$ is definable in $N [ D \backslash \{ x_{0} \} ]$ and so, by ABSP as just noted above, is less than $x_{0}$. The Claim follows. $\square$

Define $\tilde{H} = {\text {SH}}^{K_{D}} [ \bar{x}_{0} \cup \overline{ D } \backslash \{ \bar{x}_{0} \} ]$. Let $\sigma : \overline{K} {\leftrightarrow }\tilde{H}$ again with $\overline{K}$ transitive. Let $\widetilde{\tau }=_{{\text {df}}}{\text {crit}} ( \sigma )$ Then:

(4) (i) $$\overline{ K} {\upharpoonright }\widetilde{\tau }= K_{D} {\upharpoonright }\widetilde{\tau }$$;

(ii) $$\widetilde{\tau }= \bar{x}_{0}$$;

(iii) $$K^{q+1} =^{*} K_{D} =^{*} \overline{ K }$$.

### Proof

(i) just follows from the definition of $\tilde{H}$ and so $\overline{ K}$. For (ii): we just need to check that $\widetilde{\tau }\le \bar{x}_{0}$; but this now follows from (3) and the isomorphism of the relevant collapse map.

For (iii): the first $=^{*}$ comes from the defining property of *p* from (2), and thence *D*. That $\overline{ K }  \le ^{*} K_{D}$ follows immediately from the fact that $\sigma : \overline{ K } {\,\longrightarrow \,}K_{D}$ is an elementary map (and the same argument from the Dodd-Jensen Lemma as that for (2)). However, by the same argument once more, also $K^{q+1} \le ^{*} \overline{ K }$ since ${\text {ran}} ( \pi ^{q+1 } )^{-1} { \,\subseteq \, } \pi ^{-1}_{D}$“${\text {ran}} \sigma$, and thus $\sigma ^{-1} \circ \pi _{D} \circ ( \pi ^{q+1} )^{-1}: K^{q+1} \longrightarrow \overline{ K }$, elementarily. $\square$

(5) $\overline{ K}  \models$“ $\bar{x}_{0}$ *is a regular cardinal*”.

### Proof

Otherwise, if $\delta =_{{\text {df}}}{\text {cf}}^{\overline{ K}} ( \bar{x}_{0} ) < \bar{x}_{0}$ and $g \in \overline{ K}$ is a witnessing cofinal map $g: \delta {\,\longrightarrow \,}\bar{x}_{0}$, we can apply $\sigma$ and have that $\delta = \sigma ( \delta ) = {\text {cf}}^{K_{D}} ( \sigma ( \bar{x}_{0} )) < \sigma ( \bar{x}_{0} )$ where, (by (4)(ii)) $\sigma ( \bar{x}_{0} ) > \bar{x}_{0}$. But this is absurd as $\sup {\text {ran}} ( \sigma (g)) = \sup {\text {ran}} (g) = \bar{x}_{0}$. $\square$

(6) $$\bar{x}_{0}$$ *is a* $$K_{D}$$*-singular.*

### Proof

$\pi _{D} ( \bar{x}_{0} ) =x_{0} \in X$ and has, by (1), *V*-cofinality $\tau _{k}$, whilst at the same time the closed $G_{n}$ is unbounded below it, where *n* is such that $x_{0} \in ( \tau _{n-1}, \tau _{n} )$; hence $x_{0} \in G_{n}$ and has $o^{K} (x_{0} ) < \tau _{k}$. By Cox’s theorem we must have $x_{0}$ a *K*-singular. Hence (6) follows by elementarity. $\square$

By (4), $K_{D} =^{*} \overline{ K}$ and a comparison iteration of these two cannot include a truncation on either side. However (5) and (6) imply that $\mathcal {P} ( \bar{x}_{0} )_{\overline{K }} \subsetneqq \mathcal {P} ( \bar{x}_{0} )_{K_{D}}$. But as the mice $K_{D}, \overline{ K}$ agree below $\bar{x}_{0}$ (by (4)(i) and (ii)), the only way to allow the power sets to become equal, is for there to be an extender $E=E_{\alpha }$ in one of the models $K^\alpha$ of the coiteration with $( {\text {crit}} ( E_{\alpha } )^{+} )^{K {\alpha }} \le \bar{x}_{0}$ whilst $\alpha \ge \bar{x}_{0}$.

Recall that as we are below $O^{{\text {\P }}}$ we have no overlapping extenders and that as $K_{D}, \overline{ K}$ agree below $\bar{x}_{0}$ we shall have unboundedly (below $\bar{x}_{0}$) initial segments of *E* on the model on the $\bar{K}$ side (by the Initial Segment Condition defining these hierarchies). But that would imply in $\bar{K}$ that ${\text {crit}} ( E )$ is strong up to the inaccessible $\bar{x}_{0}$. (Note that $\pi _{D}^{-1}\circ \sigma : \bar{K} {\,\longrightarrow \,}\mathcal {K}$ and $\pi _{D}^{-1}\circ \sigma (\bar{x} _{0})= \tau _{q}$. Hence $\bar{x}_{0}$ is a $\bar{K}$-inaccessible.) Moreover we have $o^{\bar{K}}(crit(E_{\alpha }))\ge \bar{x}_{0}$. Hence in *K* by elementarity we have

$$o^{K}(\pi _{D}^{-1}\circ \sigma ({\text {crit}}(E_{\alpha })) \ge \tau _{q}.$$

But $q\in Q$ and so there is no such extender $E_{\alpha }$. Hence the conjunction of (4), (5) and (6) in this case (i) leads to a contradiction. $\square$

### Proof

(ii). Suppose the conclusion in *K* fails: then in this case we could define for our *Q* an infinite set of $m<\omega$ so that for every $\mu < \tau _{m}$ there is $\delta _{\mu } < \tau _{m}$ so that for no $\delta > \delta _{\mu }$ do we have ${\text {crit}} ( E_{\delta } ) = \mu$. We may run the proof of (i) (removing now the sets $G_{m}$ from our argument), and we should conclude (4) and (5) as before. (6) also holds since we are now assuming that ${\text {cf}} ( x_{0} ) = {\text {cf}}^{K} ( x_{0} ) = \tau _{k}$. So $\bar{x}_{0}$ is singular in $K_{D}$ by elementarity. But now we may reach a contradiction in the same manner: there can be no extender $E_{\alpha }$ as there with $\mu =_{{\text {df}}}{\text {crit}} ( E_{\alpha } ) < \bar{x}_{0}$ with $\alpha > \bar{x}_{0}$, in order to evade having to take a truncation in the comparison of $K_{D}$ with $\overline{ K}$. Hence our original supposition is false and so for all sufficiently large *m* we have some $\lambda _{m} < \tau _{m}$ with $o^{K} ( \lambda _{m} ) \ge \tau _{m}$. $\square$

### Proof

(iii) Again we suppose the conclusion false; now we define an infinite set $Q { \,\subseteq \, } \omega$ of *m* for which there is $\delta _{m} < \tau _{m}$ with no $\alpha > \delta _{m}$ having ${\text {crit}} ( E_{\alpha } ) < \lambda _{m}$. Note that the $\delta _{m}$ are definable in $\mathcal {K}$ from the $\tau _{m}$, and consequently are less than $x_{m}$. We may run the proof of (i) (again removing the sets $G_{m}$ from our argument). (4) and (5) as before. Now at (6): we have $x_{0}$ is singular in *K* (as it is between $\max \{ \delta _{q}, \lambda _{q} \}$ and $\tau _{q} = \lambda _{q}^{+K}$); hence $\bar{x}_{0}$ is so in $K_{D}$. The contradiction is now the same: we cannot truncate on either side, but neither do we have an extender $E_{\alpha }$ in a model with ${\text {crit}} ( E_{\alpha } ) < \pi _{q} ( \lambda _{q} )$ for an $\alpha \ge \bar{x}_{0} > \pi _{q} ( \delta _{q} )$. $\square$

The following is just a reformulated consequence of the above.

### Corollary 1

$( {\text {ZFC}} )$ $(\lnot O^{\P })$ ${\text {ABSP}} \text{ for } \text{ some } \text{ infinite } \text{ sequence } \text{ of } \, {\langle }\aleph _{m_{n}}{\rangle }_{n} \Rightarrow \{ o^{K} ( \alpha ) \mid \alpha < \aleph _{\omega } \}$ is unbounded in $\aleph _{\omega }$.

## Open questions

Q1 *Does *${\text {AFSP}}$ *at *$\aleph _{\omega }$ *imply directly for measurables in K* *the same consequences as* ${\text {ABSP}}$?

The template of the argument to come in the proof of Theorem 5 would work correctly if one could argue that there was an infinite free subset $X= \{ x_{m} \}_{m< \omega }$ for the internally approachable *N* for which property (3) in the proof of Theorem 5 holds. This property strengthens freeness so as to have a kind of weak ‘goodness’ property sometimes enjoyed by full indiscernibles. Koepke in [3] indeed shows that given an infinite free set for ${\mathcal {K}}$, by a kind of exchange principle argument one can replace the set by another set $\{ x'_{m} \}_{m< \omega }$ for which property (3) would hold, which he then uses (as did we in $(*)$). However, *prima facie*, there seems to be no reason in his argument why the cofinality of the $x_{m}$ would be preserved under this exchange, and, for example, ${\text {cf}}^{V} ( x_{m}' ) = \omega$ does not seem to be ruled out whilst ${\text {cf}}^{V} ( x_{m}' ) = \omega _{k}$ is what is needed in Theorem 5.

The authors of [1] also ask the following question.

Q2 Is the following consistent: For an ascending sequence of cardinals $\langle \kappa _{n} \rangle _{n}$ with supremum $\kappa$, for an arbitrary $\theta > \kappa$, and for every internally approachable $N \prec {\langle }H ( \theta ), \vec {f}, \vec {R}, \langle \kappa _{n} \rangle _{n} \ldots {\rangle }$, we have that for any $F \in N$, $F: \,^{k} \kappa {\,\longrightarrow \,}\kappa$, that for any $i_{0}< \cdots<i_{k} < \omega$, with $F ( \chi _{N} ( \kappa _{i_{1}} ), \cdots , \chi _{N} ( \kappa _{i_{k}} ) ) < \chi _{N} ( \kappa _{i_{0}} )$ then this implies that $F ( \chi _{N} ( \kappa _{i_{1}} ), \cdots , \chi _{N} ( \kappa _{i_{k}} ) ) \in N$.

Here then, applying a function to a sequence of the characteristic points, if the function takes a value less than a smaller characteristic point, then that value is not new, it is already in *N*.

Q3 Which of the two hypotheses, $o ( \kappa ) = \kappa ^{++} +1$, or that there are unboundedly many $\kappa _{m}$ with $o ( \kappa _{m} ) = \kappa _{m}^{++}$, is the exact consistency strength needed for Theorem 5 (iii)?

### Remark

Ben-Neria has stated (private communication) that the conclusion of the (iii) in this theorem is almost optimal, in that starting with a model of $o ( \kappa ) = \kappa ^{++} +1$, one can force ${\text {ABSP}}$ for an $\omega$-sequence of cardinals that are *K*-successors.
